# Dietary for the Diabetic

**Published:** 1885-09

**Authors:** 


					﻿Dietary for tlie Diabetic.
----- •
MAY EAT
Butcher’s meat of all kinds, except liver, ham, bacon, or other
smoked, salted, dried or cured meats; poultry, game, shell-fish and
fish of all kinds—fresh, salted or cured—animal soups not thickened,
beef tea and broths, the almond, bran, or substitute for ordinary
bread, eggs dressed in any way, cheese, creem cheese, butter, cream,
greens, spinich, *turnip-tops, *French beans, *brussels sprouts,
* cauliflower, *broccoli, *cabbage, *asparagus, Seakale, Vegetable
marrow, mushrooms, water cress, mustard and cress, cucumber, let-
tuce, endive, radishes, celery, vinegar, oil, pickles, jelly, flavored but
not sweetened; savory jelly, blanch-mange, made with cream and not
milk ; custard, made without sugar; nuts of any description, except
chestnuts; olives.
MUST AVOID EATING
Sugar in any form, wheaten bread and ordinary biscuits of all
kinds, rice, arrowroot, sago, tapioca, maccaroni, vermicelli, potatoes,
carrots, parsnips, beet-root, peas, Spanish onions, pastry and puddings
of all kinds, fruits of all kinds, fresh and preserved.
MAY DRINK
Tea, coffee, cocoa from nibs, dry sherry, claret, dry sauterne, burgundy,
chablis, hock, brandy, and spirits that have not been sweetened, soda-
water, Burton bitter ale, in moderate quantity.
MUST AVOID DRINKING
Milk, except sparingly, sweet ales, mild and old, porter and stout,
cider, all sweet wines, sparkling wines, port wine, unless sparingly,
liquors.—Therapeutic Gazette.
Notice.—Those marked with an asterisk (♦) may only be eaten in moder-
ate quantity, and should be boiled in a large quantity of water.
				

